# Farmers’ Perceptions of Local Food Procurement, Mississippi, 2013

**DOI:** 10.5888/pcd11.140004

**Published:** 2014-06-26

**Authors:** Nathan Rosenberg, Nhan L. Truong, Tyler Russell, Deja Abdul-Haqq, June A. Gipson, DeMarc A. Hickson, for the Project CHANGE Leadership Team

**Affiliations:** Author Affiliations: Nathan Rosenberg, Tyler Russell, Delta Directions, Clarksdale, Mississippi; Nhan L. Truong, Deja Abdul-Haqq, Center for Research, Evaluation and Environmental and Policy Change, My Brother’s Keeper, Inc, Jackson, Mississippi; June A. Gipson, Center for Community-Based Programs, My Brother’s Keeper, Inc, Ridgeland, Mississippi.

## Abstract

We sought to understand the experiences and perceptions of food producers regarding food procurement programs for local institutions. A total of 72 (45%) Mississippi fruit and vegetable growers completed a mailed survey, and of those that reported selling to local businesses and institutions (54%), few were selling to schools (13%). The primary motivations to sell to institutions were to increase profits (67%) and to improve nutrition within their communities (57%), while the most commonly reported barrier was a lack of knowledge about how to sell to institutions (39%). Farm to institution programs must develop evidence-based practices designed to address barriers to producers’ participation in local institutional food procurement programs.

## Objective

Food procurement from local farmers by local institutions (F2I) is becoming increasingly widespread. In the United States, the most well-known type of F2I, farm to school (F2S), has quadrupled over the past decade (1,2). F2S programs, including national model programs such as the Green Mountain Farm to School project in Vermont, promote health and improve children’s access to fruits and vegetables by linking schools to locally grown foods (3,4). In Mississippi, more than 15% of school districts reported having a F2S program in 2012 compared with none in 2010 (5,6). As programs for local institutional food procurement expand, it is important to understand the barriers to and motivations for food producers’ participation in F2S programs. We describe a study of Mississippi growers’ perceptions of, barriers to, and motivations for participation in local institutional food procurement programs.

## Methods

We used a cross-sectional survey design to assess the needs of Mississippi fruit and vegetable producers, including the experiences and perceptions of food producers in local institutional food procurement programs in Mississippi. This work was supported in part by an award to My Brother’s Keeper, Inc (MBK), from the Community Transformation Grant Program, a national effort of the Centers for Disease Control and Prevention to implement community-level chronic disease prevention interventions (7). The survey of Mississippi fruit and vegetable growers was developed and administered by a team of investigators from Delta Directions and MBK. The team vetted the survey sampling, survey construction, and response formats. The survey was refined by conducting pilot surveys with 11 local food producers and food procurement experts. The survey contained questions related to food production and safety and food producers’ perceived barriers to and motivations for participating in local institutional food procurement programs. A copy of the survey is in the Appendix.

We obtained contact information for Mississippi fruit and vegetable growers from grower cooperatives and advocacy organizations. We also found grower contact information using 2 websites: Mississippi MarketMaker (http://ms.marketmaker.uiuc.edu/) and Local Harvest (http://www.localharvest.org). In total, 159 fruit and vegetable growers were identified and mailed a survey during March 2013 through July 2013. In an introductory letter, growers were informed that completing the survey signified their consent to participate in the survey and to allow us to use the collected information in reports, publications, or presentations. Growers were also informed that their personal contact information would not be used in reports, publications, or presentations and would not be shared with anyone seeking to contact local growers. Growers were asked to return the completed survey in the provided stamped, self-addressed envelope. The MBK Institutional Review Board approved the use of these data for this study. Descriptive statistics, including proportions for categorical variables, were generated in SPSS version 21 (IBM Corp, Armonk, New York).

## Results

A total of 72 (45%) growers returned the survey. Most participants (54%) reported owning less than 50 acres of land, and 28% reported having some type of food safety certification (eg, US Department of Agriculture’s Good Agricultural Practices and Good Handling Practices). More than three-fourths of growers primarily sold food locally through either direct-to-consumer sales such as farmers markets (61%) or on-farm sales (28%), or intermediate marketing venues, such as local restaurants, institutions, or stores (49%).

Most growers who reported selling to local businesses and institutions sold directly to noninstitutional local purchasers such as restaurants (69%) and grocery stores (51%); fewer sold to local schools (13%) (Table). None of the growers reported selling directly to other institutions such as hospitals, institutions of higher education, or prisons. Of the 33 respondents who had not previously sold to local institutions, 8 (24%) indicated that they were interested in doing so. The chief barriers identified by growers for not selling to institutions was a lack of knowledge about how to sell to schools and other institutions (39%) and crops not being ready for harvesting during the academic school year (24%). Other barriers included low prices paid by schools and other institutions, insurance costs, and an imbalance between supply and demand.

**Table:** Proportion of Mississippi Fruit and Vegetable Growers (n = 72) Selling to Local Institutions and Businesses and the Perceived Barriers Among Growers Who Do Not Currently Sell to Local Institutions or Businesses, 2013

Participation or barrier	n (%)
**Participate in local food procurement programs**	39 (54)
Schools	5 (13)
Other institutions such as universities and hospitals	0
Local restaurants	27 (69)
Local grocery stores	20 (51)
Convenience stores	2 (5)
**Barriers to participation**	33 (46)
Not sure how to do it	13 (39)
Price paid is not high enough	4 (12)
Can make more money selling elsewhere	1 (3)
My crops aren’t ready during the school year	8 (24)
Schools want graded size; I can’t provide	2 (6)
Quantities wanted are too small	2 (6)
Quantities wanted are too large	3 (9)
Costly insurance	2 (6)

Overall, growers were motivated to sell to local institutions out of a desire to increase profits (67%), improve nutrition in their community (57%), increase awareness of agricultural practices among students and nongrowers (46%), support their local community (44%), and exercise good public relations (38%). Most growers also indicated that they would be interested in either having students visit their farm as part of a school field trip (58%) or visiting a classroom themselves to discuss agriculture or the importance of local food with school-aged children (57%).

## Discussion

Most Mississippi fruit and vegetable growers sell their food locally, although mainly at local farmers markets and to restaurants and grocery stores. Our findings were consistent with those from other studies (8,9) that documented perceived barriers to selling to schools and other institutions to be the small amount paid by institutions, the quantities demanded by institutions (ie, either too small or too large), and costly insurance premiums. Our findings extend the current literature by identifying additional perceived barriers: 1) misalignment of crop harvesting with the academic school year and 2) lack of knowledge about how to sell to local schools and other institutions. Growers were interested in selling to local institutions for economic and social reasons, including increased profits and improving community nutrition, a finding similar to those of previous studies (4,8–11).

Further expansion of F2I could have a substantial effect on health and nutrition in Mississippi. A review article reported that F2I programs in K through 12 schools increase the amount of fruits and vegetables consumed by students in the cafeteria, classroom, and at home; they also increase students’ knowledge about healthy eating (12). Although our survey was limited to Mississippi growers, these findings suggest that future policy and programs to expand F2I must identify and address farmers’ perceived barriers to participation if local food procurement initiatives are to reach their full potential. Policy recommendations include instituting a mini-grant program, providing tax incentives for growers who sell products to local institutions, establishing a statewide F2S week, ensuring that schools can impose a geographic preference when purchasing local food products, and creating a statewide F2I program charged with providing farmers with technical assistance and training (5,13). F2I advocates, such as the Mississippi Food Policy Council, are seeking to address the knowledge gap among farmers through informal outreach, a statewide training conference, regional networking workshops, and selling guides for farmers. These efforts should be studied carefully, allowing evidence-based methods to be developed and replicated elsewhere.

## Appendix. Mississippi Fruit and Vegetable Growers Survey

This file is available for download as an Adobe Acrobat Reader document. [PDF – 554k].
