# Supplementary material for: Farmers’ Perceptions of Local Food Procurement, Mississippi, 2013
Source: Prev Chronic Dis. 2014 Jun 26;11:E106. doi: 10.5888/pcd11.140004 (PMC4075486; doi:10.5888/pcd11.140004)
Supplement: Supplementary file 1 [file 14_0004_01.pdf]

Mississippi Fruit and Vegetable Growers Survey

Name: ____

Name of Farm (If applicable):____

Farm Address:____

____

Mailing Address (if different from farm address): _____________________________

____

Phone number:____

Email address:____

SECTION I: INFORMATION ABOUT YOUR FARM

1. Which fruits and vegetables does the farm produce?

<u>Vegetables:</u>

☐Beans, Green
☐Beans, Pole
☐Beans, Butter
☐Broccoli
☐Cabbage
☐Cauliflower
☐Corn, Sweet
☐Cucumbers
☐Eggplants
☐Greens, Collards
☐Greens, Mustard
☐Greens, Turnip
☐Kale
☐Kohlrabi
☐Okra
☐Peas, English
☐Peas, Southern
☐Peppers, Bell
☐Peppers, Hot
☐Potatoes, Irish
☐Potatoes, Sweet
☐Pumpkins
☐Squash, White
☐Squash, Winter
☐Squash, Yellow
☐Squash, Zucchini
☐Tomatoes
☐Other:
____
____

Fruits:

☐Apples

☐Blueberries

☐Cantaloupes

☐Plums

☐Strawberries

☐Other: ______________________

2. Do you use any techniques or equipment to extend your growing season?

☐ Yes

☐ No

☐ I'm not sure

3. If yes, are you interested in further extending your growing season or extending your growing season for additional crops? If you answered "no" to question 2, please skip this question.

☐ Yes

☐ No

☐ I'm not sure

4. If no, would you like to extend your growing season? If you answered "yes" to question 2, please skip this question.

☐ Yes

☐ No

☐ I'm not sure

5. Do you currently produce, or are you interested in producing, animal based products?

☐ Yes

☐ No

☐ I'm not sure

6. What is your total farm acreage?

☐ Under 10 acres

☐ 10 to 49 acres

☐ 50 to 69 acres

☐ 70 to 99 acres

☐ 100 to 139 acres

☐ 140 to 179 acres

☐ 180 to 219 acres

☐ 220 to 259 acres

☐ 260 to 499 acres

☐ 500 to 999 acres

☐ 1,000 to 1,999 acres

☐ 2,000 acres or more

7. What acreage of your farm is specifically for fruits and vegetables?

8. How many years of farm experience do you have?

☐ 1-5 years

☐ 6-10 years

☐ 11-20 years

☐ 21-35 years

☐ 36 years or more

9. Do you have any food safety certifications for your farm, such as Good Agricultural Practices (GAP)?

☐ Yes

☐ No

10. Are you interested in learning more about food safety and food safety certifications?

☐ Yes

☐ No

11. What were the most popular products sold from your farm in 2012?

1. ____

2. ____

3. ____

12. Are you interested in expanding your farming operation?

☐ Yes

☐ No

☐ I'm not sure

13. What barriers are there to future farm expansion? Check all that apply.

☐ None

☐ Location

☐ Labor availability

☐ Lack of consumer demand

☐ Regulatory barriers

☐ Lack of financial resources

☐ Zoning or land use restrictions

☐ Availability of farmers markets

☐ Competition from other farm outlets

☐ Competition from non-farm outlets

☐ Future of farm uncertain

☐ Other: ___________________________________

14. Are you familiar with Mississippi's Emerging Crops Fund?

☐ Yes

☐ No

☐ I'm not sure

If "no" or "I'm not sure," please skip to question 17.

15. Have you ever obtained a loan through the Emerging Crops Fund?

☐ Yes

☐ No

☐ I'm not sure

16. If so, did you take the loan out through a bank or directly through the fund?

____

____

17. Are you interested in programs that could help preserve your land as farmland after you retire?

☐ Yes

☐ No

☐ I'm not sure

18. Which of the following do you use to communicate when conducting farm related business? Check all that apply.

☐ Landline telephone

☐ Mobile phone

☐ Email

☐ Fax

☐ Scanner

☐ Postal mail

SECTION II: MARKETING

19. Does your farm do direct marketing? "Direct marketing" refers to sales that occur between farmers and consumers without any intermediaries such as a food distributor or processor. Common direct marketing venues include roadside markets and farmstands, CSAs, direct sales to restaurants, local institutions and stores, and pick-your-own sales.

☐ Yes

☐ No

If "no," please skip to question 26.

20. If you sell directly to consumers, how do you get your products to the consumer? Check all that apply.

☐ Farm store

☐ Delivery routes

☐ Farmers market

☐ Pick your own

☐ Roadside stand

☐ Mail order/internet sales

☐ Community supported agriculture (CSA)

☐ Direct sales to restaurants, institutions and stores

☐ Other: ______________________________

21. What months is your farm in operation for direct marketing?

22. Plans for change or improvement of direct marketing operation over the next 5 years:

☐ Expand operations

☐ No change

☐ I'm not sure

☐ Reduce operations

☐ Eliminate operation

23. Direct marketing is

☐ Essential to the operation

☐ Important to the operation

☐ Not important to the operation

24. What percentage of farm sales are wholesale compared to direct: (ex: 70% direct 30% wholesale)

____% Direct

____% Wholesale

25. Which of the following are reasons why you choose to or would choose to sell directly to consumers?Check all that apply.

☐ Extra income for operation

☐ Social contact with consumers

☐ Good location available

☐ Economic necessity

☐ Insufficient land or production

☐ Inherited the farm

☐ Other:____

26. Do you sell your products directly to any of the following? Check all that apply.

☐ Schools

☐ Other institutions, such as universities and hospitals

☐ Local restaurants

☐ Local grocery stores

☐ Convenience stores

27. If you have sold to a local institution, such as a school, university, hospital, or prison, are you interested in increasing the amount of products you sell directly to them?

☐ Yes

☐ No

☐ I have not sold to a local institution

28. If you have not sold to a local institution, would you be interested in trying to sell your products to one?

☐ Yes

☐ No

☐ I have sold to a local institution

29. If you do sell directly to a local institution, which products do you sell?

30. If you are not selling to schools or other institutions, why not?Check all that apply.

☐ I am selling to schools

☐ Not sure how to do it

☐ Price paid not high enough

☐ Can make more money selling elsewhere

☐ My crops aren't ready during the school year

☐ Schools want graded size; I can't provide

☐ Quantities wanted are too small

☐ Quantities wanted are too large

☐ I would like to

☐ Currently meeting sales demand

☐ Other: ____

____

31. Would you be interested in having students visit your farm as a part of a school field trip or educational activity?

☐ Yes

☐ No

32. Would you be interested in visiting a classroom to discuss agriculture or the importance of local food with school children?

☐ Yes

☐ No

33. If you are not selling to a local farmers market, why not? Check all that apply.

☐ Travel expenses

☐ Can make more money selling elsewhere

☐ Too much time/effort involved

☐ Farmers market rules are too strict

☐ No farmers market near my farm

☐ Currently meeting sales demand

☐ Local and/or state regulations

☐ Not enough customers/demand at the farmers market

☐ Other: ____

____

34. What would motivate you to sell food to local schools or other institutions? Check all that apply.

☐ Increase awareness of agricultural practices among students and non-farmers

☐ Economic benefit for your farm

☐ Good public relations

☐ Support local community

☐ Improve nutrition in your community

☐ Other: ____

35. Would you be able to prepare your products for a school, retail store, etc.? (i.e. slice watermelons, cut carrots, shred lettuce)

☐ Yes

☐ No

Comments: ____

____

36. Please list any concerns you may have regarding selling to a local school

____

____

Thank you for taking time to fill out this survey. Your participation is important to us.

If you have any additional comments regarding this survey, feel free to write them here.

____

____

____

____
